# A collateral circulation in ischemic stroke accelerates recanalization due to lower clot compaction

**DOI:** 10.1371/journal.pone.0314079

**Published:** 2024-11-19

**Authors:** Sandra Thalerová, Andrea Vítečková Wünschová, Patrícia Kittová, Lucie Vašátková, Michaela Pešková, Ondřej Volný, Anna Mac Gillavry Danylevska, Jan Víteček, Lukáš Kubala, Robert Mikulík

**Affiliations:** 1 International Clinical Research Center, St. Anne’s University Hospital Brno, Brno, Czech Republic; 2 Department of Biophysics of Immune System, Institute of Biophysics of the Czech Academy of Sciences, Brno, Czech Republic; 3 Department of Biochemistry, Faculty of Science, Masaryk University Brno, Brno, Czech Republic; 4 Department of Anatomy, Faculty of Medicine, Masaryk University Brno, Brno, Czech Republic; 5 Department of Biochemistry, Faculty of Medicine, Masaryk University Brno, Brno, Czech Republic; 6 Department of Histology and Embryology, Faculty of Medicine, Masaryk University Brno, Brno, Czech Republic; UCSF: University of California San Francisco, UNITED STATES OF AMERICA

## Abstract

Collaterals improve recanalization in acute ischemic stroke patients treated with intravenous thrombolysis, but the mechanisms are poorly understood. To investigate it, an *in vitro* flow model of the middle cerebral artery was developed with or without collaterals. An occlusion was achieved using human blood clots. Recanalization time, thrombolysis (clot length decrease and red blood cell (RBC) release), pressure gradient across the clot and clot compaction were measured. Results showed that with or without collateral alteplase-treated RBC dominant clots showed recanalization time 98±23 min vs 130±35 min (difference 32 min, 95% CI -6-58 min), relative clot reduction 31.8±14.9% vs 30.3±13.2% (difference 1.5%, 95% CI 10.4–13.4%) and RBC release 0.30±0.07 vs 0.27±0.09 (difference 0.03, 95% CI 0.04–0.10). Similar results were observed with fibrin-dominant clots. In RBC dominant clots, the presence vs absence of collateral caused different pressure gradients across the clot 0.41±0.09 vs 0.70±0.09 mmHg (difference 0.29 mmHg, 95% CI -0.17–0.41 mmHg), and caused the reduction of initial clot compaction by 5%. These findings align with observations in patients, where collaterals shortened recanalization time. However, collaterals did not increase thrombolysis. Instead, they decreased the pressure gradient across the clot, resulting in less clot compaction and easier distal displacement of the clot.

## Introduction

The intravenous thrombolysis using alteplase (recombinant tissue plasminogen activator, rt-PA) and the endovascular therapy (mechanical thrombectomy) represent theapproved treatment methods for ischemic stroke [1–3]. A major benefit of intravenous thrombolysis is a very simple application with relatively low cost. Despite this benefit, the thrombolytic efficacy of alteplase is limited mostly due to the fact that a large proportion of patients (up to 60–70%) [4–6] do not achieve complete recanalization. Because early recanalization is such a strong predictor of good clinical outcome [7, 8], it is critical to understand mechanisms that influence the recanalization efficacy of thrombolytics.

The recanalization achieved by alteplase includes a complex interplay between thrombolysis and mechanical degradation of the clot. Pharmacological thrombolysis affects the status of fibrin fibres within a clot and makes the clot softer. However, this effect is highly dependent on the transport of alteplase inside the clot which can be driven by diffusion (~ 1 mm h^-1^) or by interstitial flow in the clot–i.e. filtration of blood plasma through clot porous matter (≥ 10 mm h^-1^) [9]. The pressure gradient across the clot that forces the interstitial flow can, however, compress the clot making it more compact and harder to be removed mechanically [10, 11].

One of the important independent determinants of clinical outcome in ischemic stroke patients is the presence of leptomeningeal collaterals. In clinical routine, leptomeningeal collaterals are assessed at baseline vessel imaging such as CT-angiography. Good collaterals were associated with improved recanalization [12–15], higher rates of a favourable outcome, and lower mortality. An explanation for such benefits is lacking.

Two hypotheses to explain the association between leptomeningeal collaterals and recanalization were postulated. The first hypothesis suggests that thrombolysis is enhanced when thrombolytics can access the clot from both sides through retrograde filling. This dual access may accelerate clot dissolution by increasing the contact area between the thrombolytic agents and the clot [10, 12, 13, 16, 17]. The alternative explanation is that collateral flow leads to a reduction of pressure gradient across the clot, hence making the clot less compact and more prone to removal either by mechanical thrombectomy or thrombolytic therapy [11]. Additionally, collaterals can improve the recanalization of a vessel occluded with lytic resistant clot [18, 19].

## Methods

This is an *in vitro* study using a middle cerebral artery (MCA) flow model with and without a collateral vessel. The model was optimized to detect the rate and time of complete recanalization of *in vitro* vessel (i.e. clot distal displacement) and to determine the measurable lytic effect of alteplase in a highly repeatable manner [20, 21] as indicated below. The MCA is the most common site of intracranial occlusions in patients with stroke [22, 23]. The MCA model was constructed according to Thalerová et al. 2021 [20] with modifications (S1 Method). Two different types of clots were prepared from human blood samples (see Blood donors below) and used to achieve an occlusion: Red blood cell (RBC) dominant clots were prepared according to Thalerová et al. 2021. [20] Fibrin dominant clots were made using Chandler loops [24] (S2 Method). The models with and without collateral were treated with or without alteplase [20] (S3 and S4 Methods). In addition to recanalization and thrombolysis the clot compaction was determined by means of image analysis (S5 Method). The velocity of interstitial flow was determined in RBC dominant clots (S6 Method).

The study was approved by Ethics Committee of the St. Anne’s University Hospital in Brno (reference number 15V/2017). All healthy volunteers who donated blood into this study provided written informed consent. The recruitment of volunteers started after 7th of March 2017 and finished by 30th of June 2019.

### Blood donors

Blood was drawn from 16 fasting healthy donors (9 males and 7 females, Caucasians). Individuals who had received acetylsalicylic acid, non-steroidal anti-inflammatory or antiplatelet drugs within 7 days before blood collection were not included.

### Measure of recanalization and lytic efficacy

The recanalization time was defined as the primary outcome of the positive effect of collateral and was measured since the experiment start for 180 min or till the clot removal. The recanalization frequency was determined as a percentage ratio of complete recanalization to the total number of samples in the given treatment group in all biological replicates. For a detailed evaluation of the thrombolysis process, clot lysis was determined by the spectrophotometric determination of RBC release into the incubation media at 575 nm and by the change of the clot length. Relative clot reduction was determined as a percentage of clot length reduction before the release of the clot from the narrowed occlusion site, without the involvement of the information about recanalization time [20, 21]. To combine recanalization time and relative clot reduction over time, the average relative clot reduction was plotted against time. For individual values, it was set to 100% at time of recanalization. A more detailed description of individual evaluation methods is provided in the S2 Note.

### Data analysis and statistics

Data are expressed as mean ± standard deviation (SD), absolute difference of means and 95% confidence intervals (95% CI) of the difference, if not otherwise indicated. Unpaired t-test was used to compare the data. Based on previous data for the model without collateral with RBC dominant clots, we expected recanalization time to be 144±30 min [20]. The effect size of collateral vessel improvement is not specified in the literature; thus, we set the threshold of effect size to 20% (i.e. 29 min). Therefore, with 80% statistical power and a two-sided level of significance of 0.05, the inferred minimum sample size was 16. All analyses were performed with GraphPad Prism version 8.4.2 software (GraphPad Software, USA).

## Results

### RBC dominant clots

**Fig 1:**
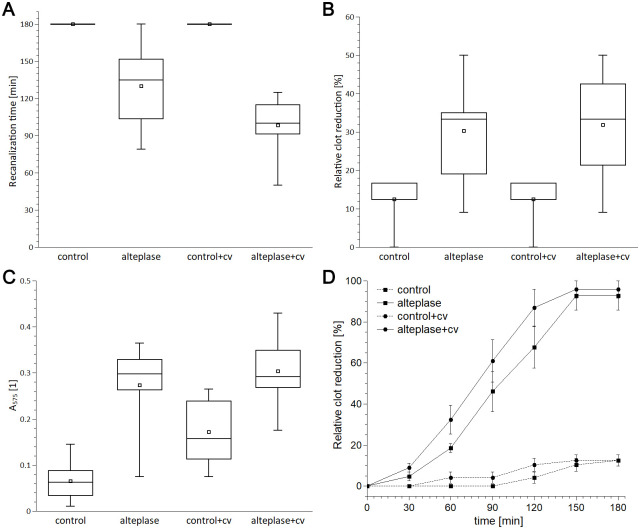
Results for RBC dominant clots: (A) time to recanalize *in vitro* model; (B) relative clot reduction; (C) RBC release; (D) relative clot degradation rate. The box plots show mean values (square), median (line), interquartile range (box), and minimum and maximum values (whiskers). The time curves show mean values (square or circle) and standard error (whiskers) of clot length at time intervals. N = 8–12. The presence of collateral augmented alteplase-induced recanalization by 25% compared to the model without collateral (A), but induced similar relative clot reduction (B), RBCs release, (C) and clot degradation rate (D). cv stands for collateral vessel.

In the model with collateral compared to the model without collateral, the treatment with alteplase resulted in lower recanalization times (98±23 min vs 130±35 min, difference 32 min, 95% CI -6-58 min). The following measurements were similar for both groups: recanalization frequency after 180 minutes 100±0% vs 75±46% (difference 25%, 95% CI 10–60%), relative clot reduction 31.8±14.9% vs 30.3±13.2% (difference 1.5%, 95% CI 10.4–13.4%), RBC release 0.30±0.07 vs 0.27±0.09 (difference 0.03, 95% CI 0.04–0.10), and clot degradation rate 0.61±0.07%/min vs 0.60±0.05%/min (difference 0.01%/min, 95% CI 0.06–0.09%/min). There was no evidence of recanalization in the control groups. Results are presented in Fig 1 and S1 Table.

### Fibrin dominant clots

**Fig 2:**
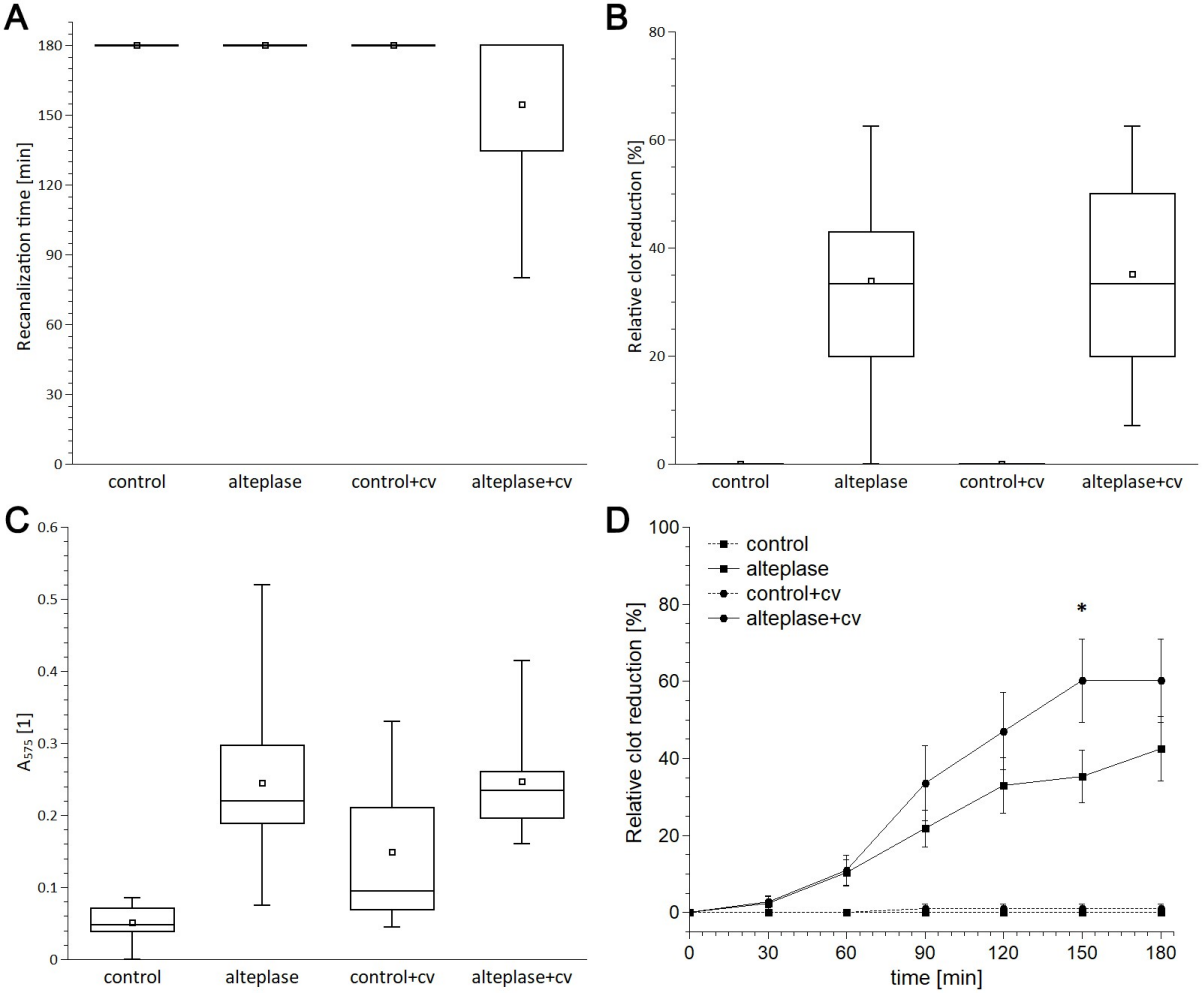
Results for fibrin dominant clots: (A) time to recanalize *in vitro* model; (B) relative clot reduction; (C) RBC release; (D) relative clot degradation rate. The box plots show mean values (square), median (line), interquartile range (box), and minimum and maximum values (whiskers). The time curves show mean values (square or circle) and standard error (whiskers) of clot length at time intervals. N = 8–13. The presence of collateral augmented alteplase-induced recanalization by 14% compared to the model without collateral (A), but induced similar relative clot reduction (B), RBCs release (C) and nearly the same relative clot degradation rate; asterisk indicates marginally significant difference between the model with and without collateral (60.2±37.5% vs 35.2±24.6%, difference 25.0%, 95% CI 1.1–51.0%), (D). cv stands for collateral vessel.

In the model with collateral as compared to the model without collateral, treatment with alteplase induced lower recanalization time (155±39 min vs 180±0 min, difference 25 min, 95% CI -1-49 min), higher recanalization frequency after 180 minutes (44±50% vs 0±0%, difference 44%, 95% CI -3-84%) and higher clot degradation rate (0.39±0.04%/min vs 0.26±0.02%/min, difference 0.14%/min, 95% CI -0.10–0.18%/min). Relative clot reduction 35.2±19.3% vs 33.9±18.7% (difference 1.3%, 95% CI 14.9–17.4%) and RBC release 0.25±0.07 vs 0.24±0.12 (difference 0.01, 95% CI 0.09–0.09) were similar for both groups. There was no evidence of recanalization in the control groups. Results are shown in Fig 2 and S2 Table.

### The combined effect of collaterals and clot type on recanalization

Alteplase-treated RBC dominant clots showed a lower recanalization time (98±23 min) compared to fibrin dominant clots (155±37 min, difference 56 min, 95% CI -28-85 min) in the model with collateral as well as higher recanalization frequency (100±0% vs 44±50%, difference 56%, 95% CI -19-94%) and clot degradation rate (0.61±0.07%/min vs 0.39±0.04%/min, difference 0.22%/min, 95% CI -0.18–0.26%/min). Overall thrombolysis, i.e. relative clot reduction (31.8±14.9% vs 35.2±19.3%, difference 3.3%, 95% CI 11.6–18.2%) and RBC release (0.30±0.07 vs 0.25±0.07, difference 0.06, 95% CI 0.01–0.12) were similar for both types of clots. Results concerning thrombolysis were almost similar in the model without collateral. Results are shown in S3 Table. These results suggest high thrombolytic resistance of fibrin dominant clots.

### The initial clot compaction

**Fig 3:**
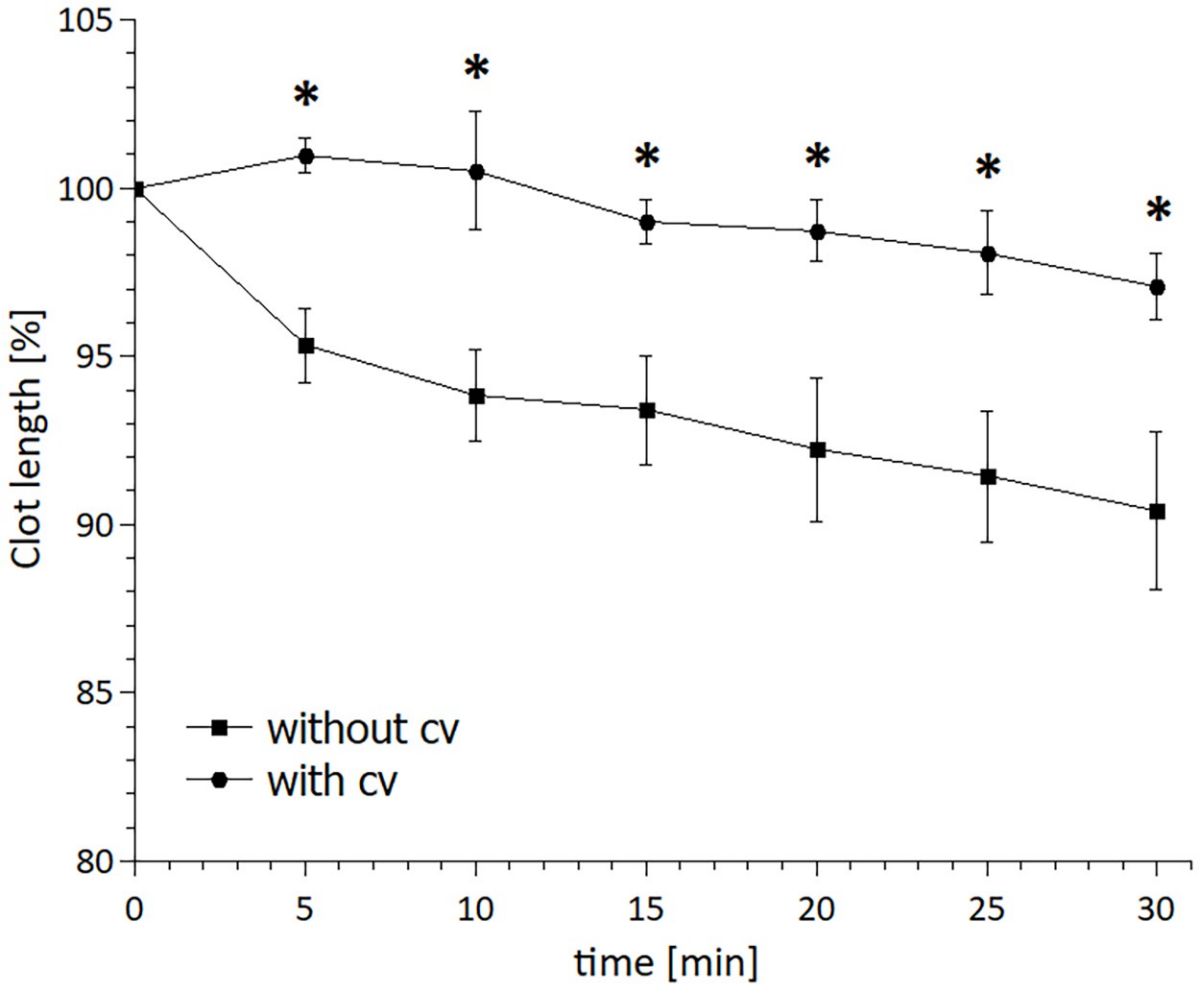
Initial RBC dominant clot compaction in the *in vitro* MCA model without and with collateral with alteplase applied at clinically relevant concentration (1.3 mg L^-1^). Clot length relative to baseline (100%) was determined by the image analysis with pictures taken every 5 min for the first 30 min of the experiment. Collateral circulation reduced the clot compaction, documented as significantly bigger clot length (about 5%) in presence of collateral after 5 minutes of the experiment start. Data are presented as mean ± S.E. of mean. N = 6. Asterisk indicates a statistically significant difference between variants at particular time. cv stands for collateral vessel.

Clots in the model without collateral compared to the model with collateral were significantly more compacted after 5 minutes of the experiment start and remained shorter for the rest of the experiment (30 min): 5 min 95.3±2.8% vs 101.0±1.2% (difference 5.6%, 95% CI -2.9–8.4%); 30 min 90.4±5.8% vs 97.1±2.4% (difference 6.7%, 95% CI -1.0–12.3%). The pressure across clot without collateral was 0.70±0.09 vs with 0.41±0.09 collateral mmHg, difference 0.29 mmHg, 95% CI 0.17–0.41 mmHg); results are presented in S4 Table. After 5 minutes, the clot length in the vessel showed a gradual decrease in both models. There was no significant difference in the rate of decrease since the linear regression slope in the model without collateral was similar to the one in the model with collateral (-0.19±0.01%/min vs -0.15±0.01%/min, difference 0.03%/min, 95% CI -0.02–0.05%/min). Results are shown in Fig 3.

### The interstitial flow in clots

The interstitial flow in RBC dominant clots showed high biological variability among clots from different blood donors: mean 43.8 mm h^-1^ mmHg^-1^, median 6.2 mm h^-1^ mmHg^-1^, lower 95% CI 8.6 mm h^-1^ mmHg^-1^, upper 95% CI 79.1 mm h^-1^ mmHg^-1^. There was no correlation between the pressure gradient and interstitial flow within clots.

## Discussion

This *in vitro* study evaluated the relationship between the recanalization seen as clot distal displacement and the presence of collateral vessel. The flow model was used to represent the most common site of intracranial occlusions, MCA [22, 23], with and without collateral circulation. Internal validity was ensured by using the therapeutic level of alteplase and two common types of human blood clots. Further, the model enabled permanent flow through the MCA bifurcation up to the occlusion site, with -time monitoring of recanalization and thrombolysis. Our model is unique as documented by literature search in the National Library of Medicine’s PubMed database (keywords: stroke, thrombolysis, *in vitro*, model and flow). Our model was able to detect the recanalization and lytic effect of alteplase in a highly repeatable manner. To demonstrate external validity, relative clot reduction of RBC dominant clots at 180 min with alteplase in the model without collateral ranged from 9.1% to 50% with median of 33%, which is similar to the median clot reduction of 32% in patients [25]. Further, observed clot degradation rate of RBC dominant clots ranged from 0.45%/min to 0.78%/min with mean value 0.61%/min, which matched relative clot change of 0.59%/min in mice [26] (S2 Note).

The first major finding of our study is that we confirmed the positive effect of collateral on recanalization in an *in vitro* model. Hence, the presence of collateral improved alteplase-induced recanalization compared to the model without collateral (lower recanalization time by 25% with RBC dominant clots and by 14% with fibrin dominant clots and higher recanalization frequency by 44% with fibrin dominant clots). Therefore, our model was able to replicate findings from previous clinical studies, thus further supporting external validity of our model. Consistent with our observation, a clinical study [12] reported early recanalization in 40% of patients with excellent collaterals, but only in 12% of patients with poor/moderate collaterals. Further, in a retrospective study focused on the relation of collateral status and recanalization [27], poor collaterals were observed in 33% of the patients without recanalization, but only in 12% of patients with recanalization. A number of clinical studies [16, 28–31] reported good collateral status as an important predictor of favourable clinical outcome in patients with MCA occlusion, without pointing to a mechanism of collateral action. Additionally, prediction model of early recanalization reported good collaterals as independent and significant predictor of early recanalization [32]. Having confirmed the positive effect of collaterals in recanalization *in vitro*, we explored two potential mechanisms behind this effect.

**Fig 4:**
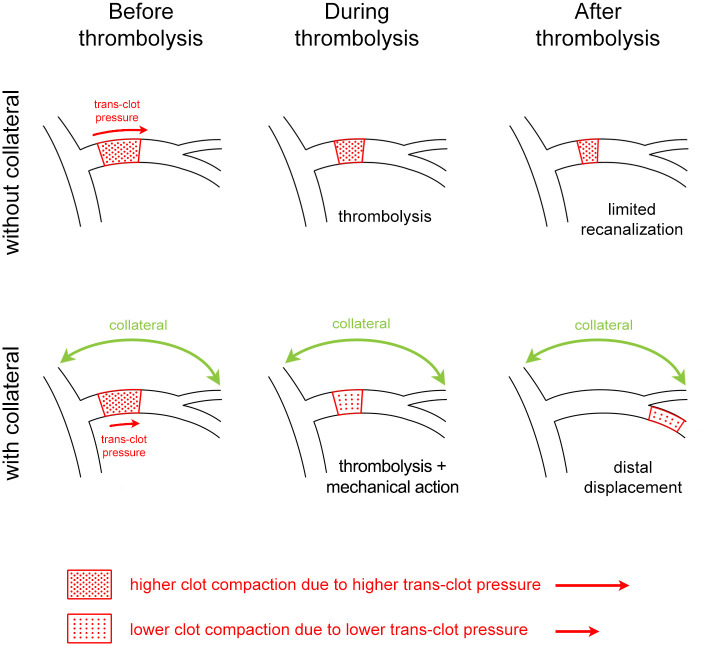
Proposed concept of collaterals action: The collateral circulation lowered the pressure across the clot which resulted in lesser clot compaction and a subsequent easier clot distal displacement. The clot occludes the MCA and is compacted due to the trans-clot pressure. This pressure is more pronounced in the MCA without collateral. The presence of collateral does not change the thrombolysis rate. The less compacted clot is more prone to the distal displacement, ie. to the faster recanalization in a primary occlusion site.

The main finding related to the mechanism of collaterals action is that clots in the model without collateral were more compacted compared to clots with collateral. Less compaction was related to lower pressure gradient (difference between proximal and distal pressure about 42%) across the clot in the presence of collateral. These findings are consistent with the results of a study that measured blood pressure in the artery proximal and distal to an occlusion in stroke patients during thrombolytic therapy [33]. They indicated that higher proximal-distal blood pressure gradient across the clot resulted in higher compaction of the clot, thus making it more difficult to remove [33]. Similarly, a study focusing on clot dynamics and migration in stroke patients suggested that lower compaction allows for easier recanalization by distal displacement of the clot [34]. Further, we focused on interstitial flow of circulating medium within the clot, because it is the major factor, which modulates thrombolysis since it introduces alteplase and plasminogen into the clot [9]. We found that the geometrical speed of the medium through the clot was about 0.0012 cm s^-1^ mmHg^-1^. Such value for RBC dominant clots prepared *in vitro* is close to data from patients ranging from 0.0043 to 0.0225 cm s^-1^ mmHg^-1^ reported in a study focusing on perviousness and permeability in acute ischemic stroke [35]. There was no apparent link between pressure gradient across the clot and interstitial flow. Such data correlated well with the same level of thrombolysis regardless of the presence of collateral. We have proven such conclusion by the same level of RBC release and relative clot reduction regardless of presence of collateral. It was observed consistently in both RBC dominant as well as fibrin dominant clots. Our model with collateral allowed for retrograde filling nevertheless no elevated thrombolysis was observed. Such observation excluded the positive effect of retrograde filling on thrombolysis. This has not been observed before, based on literature search in the National Library of Medicine’s PubMed database (keywords: stroke, collateral, retrograde filling, *in vitro*). Collectively, the data clearly support the hypothesis by Nogueira *et al*. [11] that the impact of collateral circulation is by pressure change making the clot less compact and the occluded vessel easier to recanalize (Fig 4). Further, the evidence from this study suggests that recanalization involves both biochemical and mechanical component, thus thrombolysis is a necessary, but not a sufficient condition of successful recanalization as indicated by a mathematical model [36].

The secondary finding is that successful removal of dense fibrin dominant clots compared to RBC dominant clots was achieved only with the combination of alteplase treatment and the presence of collateral. Whereas with RBC dominant clots, recanalization was achieved by the action of alteplase alone, and the presence of collateral further improved recanalization. Hence, clot composition and structure had an impact on recanalization rate of *in vitro* vessel. According to the literature, fibrin dominant clots are more resistant removal [37–39]. Therefore, our data suggest that resistance of some clots, such as fibrin dominant clots, to thrombolysis could be overcome by presence of collateral. Such data are in line with clinical observations where arteries occluded with resistant clots were easier to be recanalized in the presence of collateral [18, 19].

A limitation of the study may arise from the use of lower flow rates compared to those observed in pathophysiological scenarios, resulting in reduced pressure gradients across clots. This simplification was necessary to balance the mechanical stability of the occlusion while preserving its susceptibility to alteplase degradation, as large occlusions are not sensitive to alteplase. However, the model effectively preserves the primary mechanism wherein collateral circulation diminishes pressure on the clot and enhances recanalization. Further, we used blood from healthy donors, which might be limitation because in clinical practice patients suffer from comorbidities. With the exception of diabetes, these comorbidities do not directly affect function of alteplase [40]. Hence, we do not expect major differences in results that would be obtained using blood from patients.

In conclusion, our data showed that, similarly as in patients, the presence of collateral vessel improved recanalization. The presence of collateral shortened recanalization time and reduced clot compaction but did not enhance clot lysis. Therefore, the influence of collateral circulation on recanalization was mediated through hemodynamic mechanisms, particularly because a collateral circulation lowered pressure across the clot, which resulted in less clot compaction and subsequent easier clot distal displacement due to partial lysis by thrombolytics (Fig 4). Additionally, we documented that presence of the collateral can overcome the natural resistance of dense fibrin dominant clots to thrombolytic treatment.

## Supporting information

S1 Method *In vitro* model. (PDF)

S2 Method Clots and plasma preparation. (PDF)

S3 Method Alteplase application. (PDF)

S4 Method Treatment groups. (PDF)

S5 Method Image analysis. (PDF)

S6 Method Determination of velocity of interstitial flow. (PDF)

S1 Fig Transparent *in vitro* MCA model preparation. (A)–iron model prepared according to human MCA anatomy with narrowing of the vessel (see indicated diameters) based on patients’ CT angiograms (n = 4); (B)–silicone form for lost element preparation; (C)–lost element prepared from gelatin; (D)–produced *in vitro* silicone model.
(TIF)

S2 Fig Detailed representation of *in vitro* MCA model dimensions. The model reflects important anatomical features and vessels’ diameters of human MCA. (A)–model without collateral, (B)–model with collateral. Narrowing represents the site of occlusion, while bifurcation enables permanent circulation of medium past the occlusion.
(TIF)

S3 Fig Schematic of *in vitro* MCA model set-up. (A)–model without collateral, (B)–model with collateral. Individual models were connected by plastic tubes to peristaltic pump with 8 channel pump head to enable permanent circulation of medium in the system. Models with collateral had an extra tube connecting the tube before the silicon model and the collateral vessel beginning within the silicon model.
(TIF)

S4 Fig Experiment course scheme. After models’ set-up, connection to the peristaltic pump and models filling, the clots are introduced. The experiment starts upon alteplase injection and collateral opening and lasts 180 minutes (experimentally optimized).
(TIF)

S5 Fig Histological analysis of *in vitro* blood clots. RBC dominant (left) and fibrin dominant (right) clots’ sections stained with hematoxylin-eosin (top) and picro-Mallory (bottom), documenting structural difference of used clot types. Hematoxylin-eosin allowed identification of fibrin/platelet aggregates (pink), RBCs (red), and nucleated cells (dark blue), whereas picro-Mallory staining selectively demonstrated the presence of fibrin (dark pink/red), RBCs (orange), and connective tissue (blue). Visualized by light microscopy, magnification 40x.
(TIF)

S1 Table Results for RBC dominant clots in the MCA model without and with collateral. The results are expressed as recanalization (recanalization frequency, recanalization time), overall thrombolysis (relative clot reduction, RBC release) and clot degradation rate at 30 min intervals. Clot degradation rate was calculated using linear regression of relative clot reduction at 30 min intervals and is expressed as slope and corresponding standard error (S.E.) and 95% confidence interval (CI) of linear regression.
(PDF)

S2 Table Results for fibrin dominant clots in the MCA model without and with collateral. The results are expressed as recanalization (recanalization frequency, recanalization time), overall thrombolysis (relative clot reduction, RBC release) and clot degradation rate at 30 min intervals. The clot degradation rate was calculated using linear regression of relative clot reduction at 30 min intervals and is expressed as slope and corresponding standard error (S.E.) and 95% confidence interval (CI) of linear regression.
(PDF)

S3 Table Comparison of different clot types in MCA model without and with collateral. Recanalization (recanalization frequency, recanalization time), overall thrombolysis (relative clot reduction, RBC release) and clot degradation rate at 30 min intervals were compared. The clot degradation rate was calculated using linear regression of relative clot reduction at 30 min intervals and is expressed as slope and corresponding standard error (S.E.) of linear regression.
(PDF)

S4 Table Comparison of pressure gradient across RBC dominant clots in the MCA model without and with collateral. (PDF)

S1 Note Histological analysis of blood clots. (PDF)

S2 Note Determination of clot length, relative clot reduction and clot degradation rate; comparison of clot degradation rate *in vitro* with *in vivo* and patients. (PDF)

S1 Video Video of *in vitro* MCA model occlusion with RBC dominant clot. The tubes were disconnected before the silicon model and a clot was introduced through a funnel.
(MP4)

S2 Video Video of real-time recanalization of *in vitro* MCA model. (A) without and (B) with collateral.
(MP4)

S1 File Complete data. (XLSX)

## Abbreviations

MCA: middle cerebral artery
PBS: physiological buffered saline
RBC: red blood cell
