# Supplementary material for: A collateral circulation in ischemic stroke accelerates recanalization due to lower clot compaction
Source: PLoS One. 2024 Nov 19;19(11):e0314079. doi: 10.1371/journal.pone.0314079 (PMC11575800; doi:10.1371/journal.pone.0314079)
Supplement: S1 Method — (PDF) [file pone.0314079.s001.pdf]

S1 Method: *In vitro* model

The *In vitro* flow model of the MCA from a transparent material with and without collateral was developed by our research team. This model reflects important anatomical features of the MCA. The bifurcation was included to enable permanent circulation of the medium behind the occlusion.

These models were prepared by a lost element method from silicone (Sylgard 184 Silicone Elastomer, Dow Corning, USA). Briefly, an iron model was embedded with silicone, and after curing the silicone cast was cut in half to remove the iron model. A prepared mould was used for lost elements preparation (15% gelatin, 7% saccharose). Finally, the lost element was immersed in silicone. After the lost element melting the model was prepared for experiments (**S1 Figure**). The iron model was prepared according to human MCA anatomy with narrowing of the vessel recapitulating human anatomy derived from four real CT angiograms. The bifurcation was included to enable permanent circulation within the model and to mimic human MCA circulation (**S2 Figure**).

Each MCA silicone model was connected by plastic pipes (internal diameter 3.1 mm) to a peristaltic pump with 8 channel pump head (Gilson Minipuls 3, Gilson, Inc., USA), (**S3 Figure**). The models with collateral vessel had an extra 3.1 mm tube connecting the tube in front of the silicon model and the collateral vessel origin within the silicon model (**S3 Figure B**). The collateral vessel was optimized for fast retrograde filling since the clinical data show very quick retrograde filling in patients with good collaterals. [1,2] Alteplase circulation through the collateral vessel fully reached the rear side of the clot within 5 minutes since its injection into the model as determined with the fluorescein coloration test. The collateral tube had a stopcock in the middle in order to control the flow enabling proper clot insertion. The average pressure gradient over the clot was determined to be 0.70±0.09 mmHg for the model without collateral and 0.41±0.09 mmHg for the model with collateral using a manometer. Such

lowered pressure gradients as compared to pathophysiological ischemic stroke scenario were utilized in order to achieve consistent recanalization times for occlusions still responsive to alteplase (it's important to note that large occlusions are not responsive to alteplase [3]). This decision was driven by the glaring absence of guidance in the current literature regarding the accurate replication of the robust adhesion of clots in the pathophysiological context of ischemic stroke.

The models were filled with 5-fold diluted human plasma before the start of the experiment. To achieve occlusion, the tubes were disconnected before the silicon model and clot was inserted through the funnel (**S1 Video**). The experiment has started when the alteplase or mock solution was injected into the tube by Hamilton syringe (Hamilton® syringe, 700 series, Hamilton, USA) before the MCA silicon model and the stopcock in the collateral vessel was opened. Each experiment lasted 180 minutes (experimentally optimized) (**S4 Figure**) or until recanalization of occluded *in vitro* model (i.e. clot distal displacement) was achieved (**S2 Video**).

1. Zhang S, Zhang X, Yan S, Lai Y, Han Q, Sun J, et al. The velocity of collateral filling predicts recanalization in acute ischemic stroke after intravenous thrombolysis. Sci Rep. 2016;6: 27880. doi:10.1038/srep27880

2. Son JP, Lee MJ, Kim SJ, Chung J-W, Cha J, Kim G-M, et al. Impact of Slow Blood Filling via Collaterals on Infarct Growth: Comparison of Mismatch and Collateral Status. J Stroke. 2017;19: 88–96. doi:10.5853/jos.2016.00955

3. Riedel CH, Zimmermann P, Jensen-Kondering U, Stingele R, Deuschl G, Jansen O. The Importance of Size: Successful Recanalization by Intravenous Thrombolysis in Acute Anterior Stroke Depends on Thrombus Length. Stroke. 2011;42: 1775–1777. doi:10.1161/STROKEAHA.110.609693
