# Supplementary material for: A collateral circulation in ischemic stroke accelerates recanalization due to lower clot compaction
Source: PLoS One. 2024 Nov 19;19(11):e0314079. doi: 10.1371/journal.pone.0314079 (PMC11575800; doi:10.1371/journal.pone.0314079)
Supplement: S2 Method — (PDF) [file pone.0314079.s002.pdf]

S2 Method: Clots and plasma preparation

Two types of structurally different human blood clots (**S5 Figure**) were employed in both (without and with collateral) MCA models, namely red blood cell (RBC) dominant and fibrin dominant. RBC dominant clots were prepared from 100 μL of whole human venous blood without anticoagulants and clotted in borosilicate glass tubes (internal diameter 6 mm) for 4 hours at room temperature to allow for proper retraction. [1] Fibrin dominant clots were prepared by optimized Chandler loop method [2] from 800 μL healthy donors' whole venous blood anticoagulated with 3.8% sodium citrate (3.8% sodium citrate and blood in ratio 1:149), supplemented with human thrombin (Tisseel Kit Fibrin Sealant, Baxter International Inc., USA) and recalcified with $CaCl_2$ to a final concentration 1 IU mL^{-1} and 12.2 mM, respectively, by clotting for 90 minutes at 37°C in silicone Chandler loops (4 mm x 6 mm, Gilson) with a diameter of 64 mm, with the rotational speed of 33 mm s^{-1}.

To document the structural differences of prepared clot types, histological analysis was carried out. A more detailed description of the histology procedure is provided in the **S1 Note**.

Plasma was freshly prepared for each experiment from citrated blood (3.8% sodium citrate and blood in standard ratio 1:9) by centrifugation (700 g, 10 min, 4°C); diluted 5-fold with physiological buffered saline (PBS), (pH 7.4, 10 mM Na_2HPO_4, 1.8 mM KH_2PO_4, 2.7 mM KCl, 137 mM NaCl) and kept at 4°C prior to the experiment.

1. Sutton JT, Ivancevich NM, Perrin SR, Vela DC, Holland CK. Clot Retraction Affects the Extent of Ultrasound-Enhanced Thrombolysis in an Ex Vivo Porcine Thrombosis Model. Ultrasound in Medicine & Biology. 2013;39: 813–824. doi:10.1016/j.ultrasmedbio.2012.12.008

2. Chandler AB, Jacobsen CD. In Vitro Thrombosis in Thrombotic and Hemorrhagic Diseases. Scandinavian Journal of Clinical and Laboratory Investigation. 1967;20: 129–139. doi:10.3109/00365516709076933
