# Supplementary material for: A collateral circulation in ischemic stroke accelerates recanalization due to lower clot compaction
Source: PLoS One. 2024 Nov 19;19(11):e0314079. doi: 10.1371/journal.pone.0314079 (PMC11575800; doi:10.1371/journal.pone.0314079)
Supplement: S3 Method — (PDF) [file pone.0314079.s003.pdf]

**S3 Method: Alteplase application**

Alteplase (Actilyse, provided by Boehringer-Ingelheim International GmbH, Germany; Z. Nr. 1-24,717) was dissolved in distilled water to a concentration of 1 mg $mL^{-1}$ and was stored aliquoted at -20°C (not re-frozen once thawed). The final concentration of alteplase was selected to be in line with clinically relevant dosing used in patients with acute ischemic stroke (1.3 mg $L^{-1}$), according to the manufacturer's instructions and supporting pharmacokinetic data. [1]

1. Acheampong P, Ford GA. Pharmacokinetics of alteplase in the treatment of ischaemic stroke. Expert Opinion on Drug Metabolism & Toxicology. 2012;8: 271–281. doi:10.1517/17425255.2012.652615
