# Supplementary material for: A collateral circulation in ischemic stroke accelerates recanalization due to lower clot compaction
Source: PLoS One. 2024 Nov 19;19(11):e0314079. doi: 10.1371/journal.pone.0314079 (PMC11575800; doi:10.1371/journal.pone.0314079)
Supplement: S4 Method — (PDF) [file pone.0314079.s004.pdf]

S4 Method: Treatment groups

Four experimental groups were established to test the hypothesis that the presence of collateral vessel enhances the alteplase-induced recanalization. These four groups included: “control” (subject without collateral, without thrombolytic treatment); “alteplase-treated” (subject without collateral, with thrombolytic treatment); “control with collateral” (subject with collateral, without thrombolytic treatment); “alteplase-treated with collateral” (subject with collateral, with thrombolytic treatment).
