# Supplementary material for: A collateral circulation in ischemic stroke accelerates recanalization due to lower clot compaction
Source: PLoS One. 2024 Nov 19;19(11):e0314079. doi: 10.1371/journal.pone.0314079 (PMC11575800; doi:10.1371/journal.pone.0314079)
Supplement: S5 Method — (PDF) [file pone.0314079.s005.pdf]

S5 Method: Image analysis

To evaluate the initial clot compaction, images were taken with a fixed stand web camera (Logitech HD Webcam C525, Logitech International S.A., Switzerland) at full HD resolution (8 MPixel) at 5-minute intervals for the first 30 minutes of the experiment. Length of clots was evaluated using a manual procedure in ImageJ software (Wayne Rasband, National Institutes of Health, USA).
