# Supplementary material for: A collateral circulation in ischemic stroke accelerates recanalization due to lower clot compaction
Source: PLoS One. 2024 Nov 19;19(11):e0314079. doi: 10.1371/journal.pone.0314079 (PMC11575800; doi:10.1371/journal.pone.0314079)
Supplement: S6 Method — (PDF) [file pone.0314079.s006.pdf]

S6 Method: Determination of velocity of interstitial flow

RBC dominant clots were carefully inserted into the 1 mL-syringe barrel (internal diameter 4.7 mm). The resulting occluded length was 6 to 12 mm. The syringe barrel was connected with the blunt end of the injection needle to a tube (internal diameter 0.38 mm). Air bubbles between the clot and the tube were avoided by filling the injection needle with PBS stained with phenol red. Clots were allowed to settle for 5 min. The experiment was started by filling up the space above the clot in the syringe barrel with PBS (liquid column 50 – 80 mm) in order to generate a pressure gradient across the clot. The interstitial flow through the clot was followed as the movement of the liquid front in the tube specified above. To standardize the data, the interstitial flow velocity at a pressure gradient of 1 mmHg mm^{-1} through the clot was calculated (considering the physical dimensions of the apparatus and the pressure generated due to the liquid column above the clot). The flow through above mentioned setup without the clot was two orders of magnitude higher than with the clot.
