# Supplementary figures and images for: A collateral circulation in ischemic stroke accelerates recanalization due to lower clot compaction
Source: PLoS One. 2024 Nov 19;19(11):e0314079. doi: 10.1371/journal.pone.0314079 (PMC11575800; doi:10.1371/journal.pone.0314079)

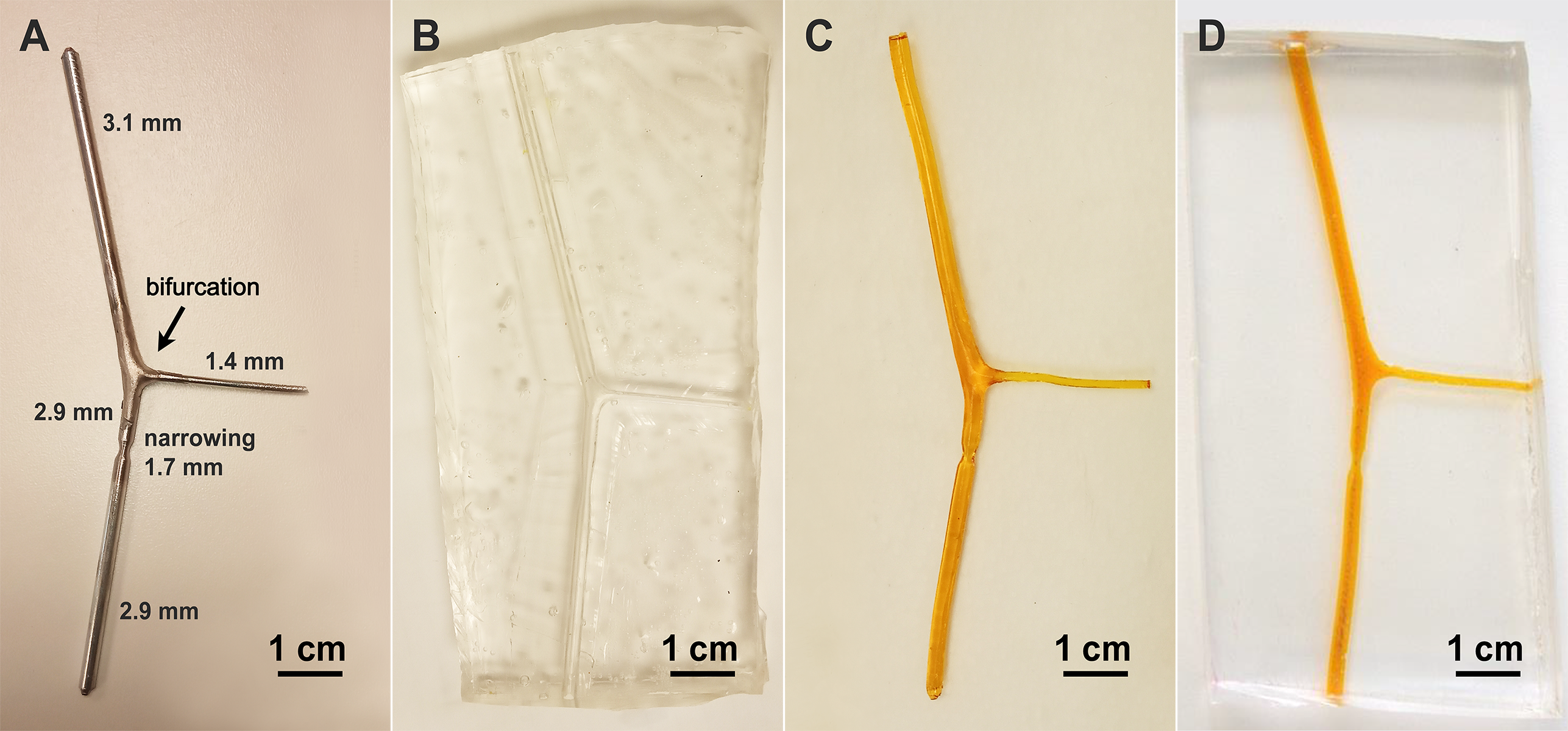

Supplement: S1 Fig — (A)–iron model prepared according to human MCA anatomy with narrowing of the vessel (see indicated diameters) based on patients’ CT angiograms (n = 4); (B)–silicone form for lost element preparation; (C)–lost element prepared from gelatin; (D)–produced in vitro silicone model. (TIF) [file pone.0314079.s007.tif]

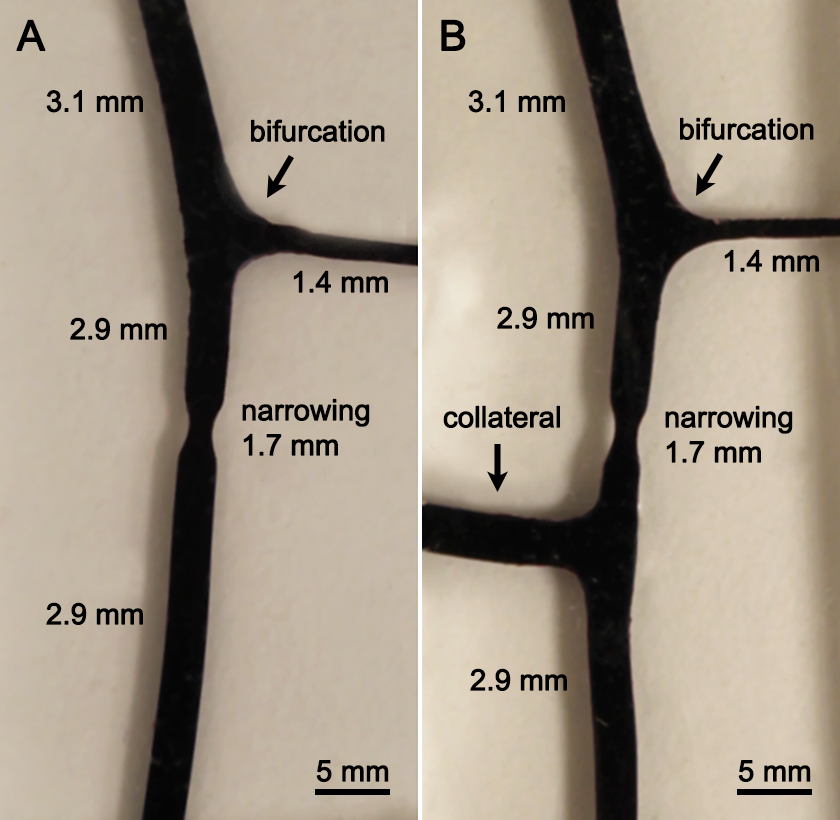

Supplement: S2 Fig — The model reflects important anatomical features and vessels’ diameters of human MCA. (A)–model without collateral, (B)–model with collateral. Narrowing represents the site of occlusion, while bifurcation enables permanent circulation of medium past the occlusion. (TIF) [file pone.0314079.s008.tif]

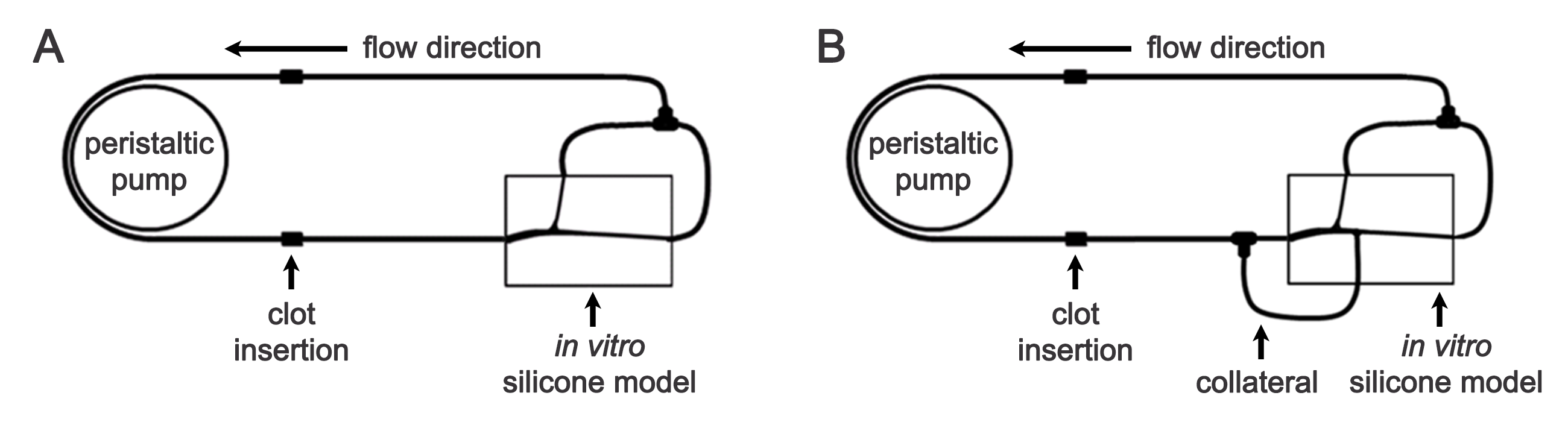

Supplement: S3 Fig — (A)–model without collateral, (B)–model with collateral. Individual models were connected by plastic tubes to peristaltic pump with 8 channel pump head to enable permanent circulation of medium in the system. Models with collateral had an extra tube connecting the tube before the silicon model and the collateral vessel beginning within the silicon model. (TIF) [file pone.0314079.s009.tif]

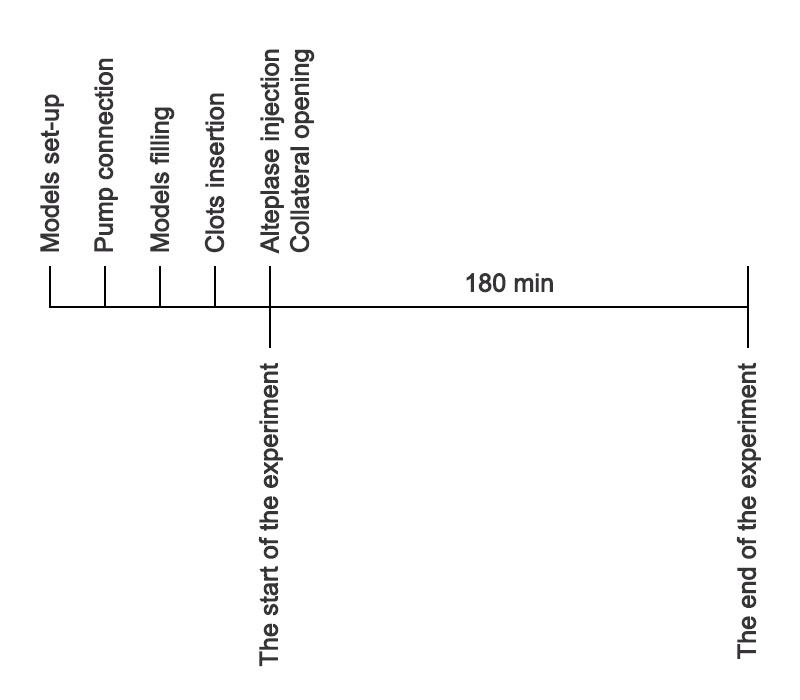

Supplement: S4 Fig — After models’ set-up, connection to the peristaltic pump and models filling, the clots are introduced. The experiment starts upon alteplase injection and collateral opening and lasts 180 minutes (experimentally optimized). (TIF) [file pone.0314079.s010.tif]

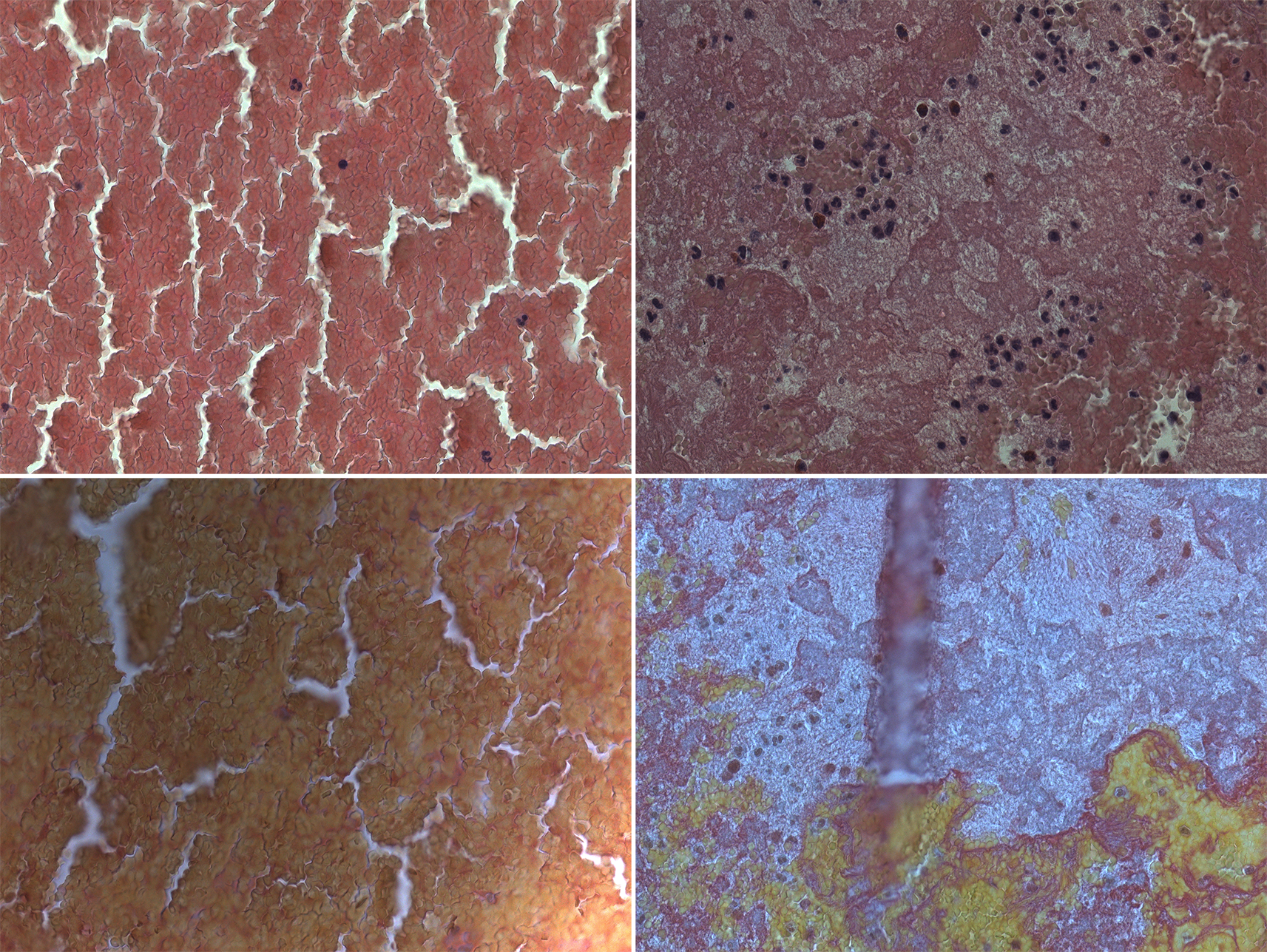

Supplement: S5 Fig — RBC dominant (left) and fibrin dominant (right) clots’ sections stained with hematoxylin-eosin (top) and picro-Mallory (bottom), documenting structural difference of used clot types. Hematoxylin-eosin allowed identification of fibrin/platelet aggregates (pink), RBCs (red), and nucleated cells (dark blue), whereas picro-Mallory staining selectively demonstrated the presence of fibrin (dark pink/red), RBCs (orange), and connective tissue (blue). Visualized by light microscopy, magnification 40x. (TIF) [file pone.0314079.s011.tif]
