# Supplementary material for: A collateral circulation in ischemic stroke accelerates recanalization due to lower clot compaction
Source: PLoS One. 2024 Nov 19;19(11):e0314079. doi: 10.1371/journal.pone.0314079 (PMC11575800; doi:10.1371/journal.pone.0314079)
Supplement: S1 Table — The results are expressed as recanalization (recanalization frequency, recanalization time), overall thrombolysis (relative clot reduction, RBC release) and clot degradation rate at 30 min intervals. Clot degradation rate was calculated using linear regression of relative clot reduction at 30 min intervals and is expressed as slope and corresponding standard error (S.E.) and 95% confidence interval (CI) of linear regression. (PDF) [file pone.0314079.s012.pdf]

Recanalization frequency [%]	Mean	Median	SD	CI (95%)	Count
Control	0	0	0	0	8
Alteplase	75	100	46	39	12
Control + cv	0	0	0	0	8
Alteplase + cv	100	100	0	15	11
**Recanalization time [min]**	**Mean**	**Median**	**SD**	**CI (95%)**	**Count**
Control	180	180	0	0	8
Alteplase	130	135	35	22	12
Control + cv	180	180	0	0	8
Alteplase + cv	98	100	23	16	11
**Relative clot reduction [%]**	**Mean**	**Median**	**SD**	**CI (95%)**	**Count**
Control	12.5	16.7	7.7	6.5	8
Alteplase	30.3	33.3	13.2	8.4	12
Control + cv	12.5	16.7	7.7	6.5	8
Alteplase + cv	31.8	33.3	14.9	9.5	12
**RBC release [1]**	**Mean**	**Median**	**SD**	**CI (95%)**	**Count**
Control	0.07	0.06	0.04	0.04	8
Alteplase	0.27	0.30	0.09	0.06	12
Control + cv	0.17	0.16	0.08	0.06	8
Alteplase + cv	0.30	0.29	0.07	0.04	12
**Clot degradation rate [%/min]**	**Slope**	**S.E.**	**CI (95%)**	**Count**	
Control	0.07	0.02	0.04	8	
Alteplase	0.60	0.05	0.14	12	
Control + cv	0.08	0.01	0.03	8	
Alteplase + cv	0.61	0.07	0.17	11	

CI, confidence interval; cv, collateral vessel; SD, standard deviation; S.E., standard error
