# Supplementary material for: A collateral circulation in ischemic stroke accelerates recanalization due to lower clot compaction
Source: PLoS One. 2024 Nov 19;19(11):e0314079. doi: 10.1371/journal.pone.0314079 (PMC11575800; doi:10.1371/journal.pone.0314079)
Supplement: S2 Table — The results are expressed as recanalization (recanalization frequency, recanalization time), overall thrombolysis (relative clot reduction, RBC release) and clot degradation rate at 30 min intervals. The clot degradation rate was calculated using linear regression of relative clot reduction at 30 min intervals and is expressed as slope and corresponding standard error (S.E.) and 95% confidence interval (CI) of linear regression. (PDF) [file pone.0314079.s013.pdf]

Recanalization frequency [%]	Mean	Median	SD	CI (95%)	Count
Control	0	0	0	0	8
Alteplase	0	0	0	0	11
Control + cv	0	0	0	0	9
Alteplase + cv	44	25	50	41	11

Recanalization time [min]	Mean	Median	SD	CI (95%)	Count
Control	180	180	0	0	8
Alteplase	180	180	0	0	11
Control + cv	180	180	0	0	9
Alteplase + cv	155	180	39	26	11

Relative clot reduction [%]	Mean	Median	SD	CI (95%)	Count
Control	0.0	0.0	0.0	0.0	8
Alteplase	33.9	33.3	18.7	11.3	13
Control + cv	0.0	0.0	0.0	0.0	8
Alteplase + cv	35.2	33.3	19.3	12.9	11

RBC release [1]	Mean	Median	SD	CI (95%)	Count
Control	0.05	0.05	0.03	0.02	8
Alteplase	0.24	0.22	0.12	0.07	13
Control + cv	0.15	0.10	0.11	0.08	9
Alteplase + cv	0.25	0.23	0.07	0.05	10

Clot degradation rate [%/min]	Slope	S.E.	CI (95%)	Count
Control	0.00	0.00	0.00	8
Alteplase	0.26	0.02	0.05	11
Control + cv	0.01	0.00	0.01	9
Alteplase + cv	0.39	0.04	0.10	10

CI, confidence interval; cv, collateral vessel; SD, standard deviation; S.E., standard error
