# Supplementary material for: A collateral circulation in ischemic stroke accelerates recanalization due to lower clot compaction
Source: PLoS One. 2024 Nov 19;19(11):e0314079. doi: 10.1371/journal.pone.0314079 (PMC11575800; doi:10.1371/journal.pone.0314079)
Supplement: S3 Table — Recanalization (recanalization frequency, recanalization time), overall thrombolysis (relative clot reduction, RBC release) and clot degradation rate at 30 min intervals were compared. The clot degradation rate was calculated using linear regression of relative clot reduction at 30 min intervals and is expressed as slope and corresponding standard error (S.E.) of linear regression. (PDF) [file pone.0314079.s014.pdf]

Recanalization frequency [%]	RBC dominant clots: mean±SD	fibrin dominant clots: mean±SD	RBC dominant vs fibrin dominant clots: difference (95% CI of the difference)
Control	0±0	0±0	N/A
Alteplase	75±46	0±0	75 (37-113)
Control+cv	0±0	0±0	N/A
Alteplase+cv	100±0	44±50	56 (19-94)
**Recanalization time [min]**	**RBC dominant clots: mean±SD**	**fibrin dominant clots: mean±SD**	**RBC dominant vs fibrin dominant clots: difference (95% CI of the difference)**
Control	180±0	180±0	N/A
Alteplase	130±35	180±0	50 (28-72)
Control+cv	180±0	180±0	N/A
Alteplase+cv	98±23	155±39	56 (28-85)
**Relative clot reduction [%]**	**RBC dominant clots: mean±SD**	**fibrin dominant clots: mean±SD**	**RBC dominant vs fibrin dominant clots: difference (95% CI of the difference)**
Control	12.5±7.7	0.0±0.0	12.5 (6.7-18.4)
Alteplase	30.3±13.2	33.9±18.7	3.6 (-9.9-17.1)
Control+cv	12.5±7.7	0.0±0.0	12.5 (6.7-18.4)
Alteplase+cv	31.8±14.9	35.2±19.3	3.3 (-11.6-18.2)
**RBC release [1]**	**RBC dominant clots: mean±SD**	**fibrin dominant clots: mean±SD**	**RBC dominant vs fibrin dominant clots: difference (95% CI of the difference)**
Control	0.07±0.04	0.05±0.03	0.01 (-0.03-0.05)
Alteplase	0.27±0.09	0.24±0.12	0.03 (-0.06-0.12)
Control+cv	0.17±0.08	0.15±0.11	0.02 (-0.08-0.12)
Alteplase+cv	0.30±0.07	0.25±0.07	0.06 (-0.01-0.12)
**Clot degradation rate [%/min]**	**RBC dominant clots: mean slope±S.E.**	**fibrin dominant clots: mean slope±S.E.**	**RBC dominant vs fibrin dominant clots: difference (95% CI of the difference)**
Control	0.07±0.02	0.00±0.00	0.07 (0.06-0.09)
Alteplase	0.60±0.05	0.26±0.02	0.34 (0.32-0.37)
Control+cv	0.08±0.01	0.01±0.00	0.07 (0.06-0.09)
Alteplase+cv	0.61±0.07	0.39±0.04	0.22 (0.18-0.26)

CI, confidence interval; cv, collateral vessel; N/A, not applicable; SD, standard deviation; S.E., standard error
