# Supplementary material for: A collateral circulation in ischemic stroke accelerates recanalization due to lower clot compaction
Source: PLoS One. 2024 Nov 19;19(11):e0314079. doi: 10.1371/journal.pone.0314079 (PMC11575800; doi:10.1371/journal.pone.0314079)
Supplement: S4 Table — (PDF) [file pone.0314079.s015.pdf]

[mmHg]	Model without cv	Model with cv
Mean	0.70	0.41
Median	0.69	0.41
SD	0.09	0.09
Lower 95% CI	0.60	0.32
Upper 95% CI	0.79	0.50
Count	6	6
Difference in means ± SEM	0.29±0.05	
95% CI of the difference	0.17-0.41	

CI, confidence interval; cv, collateral vessel; N/A, not applicable; SD, standard deviation; S.E., standard error
