# Supplementary material for: A collateral circulation in ischemic stroke accelerates recanalization due to lower clot compaction
Source: PLoS One. 2024 Nov 19;19(11):e0314079. doi: 10.1371/journal.pone.0314079 (PMC11575800; doi:10.1371/journal.pone.0314079)
Supplement: S1 Note — (PDF) [file pone.0314079.s016.pdf]

S1 Note: Histological analysis of blood clots

To document structural difference of used blood clot types (RBC dominant and fibrin dominant), histological analysis was carried out. Prepared clots were washed with PBS and immediately incubated in 3% glutaraldehyde in 0.1M sodium cacodylate buffer for 72 hours (at room temperature for the first 3 hours, followed by incubation at 4°C). Next, the samples were washed again, dehydrated with increasing ethanol series, and embedded in paraffin and cut into 2 µm sections. Individual sections were mounted on microscopic slides, and after the removal of paraffin with xylene and rehydration, samples were stained with hematoxylin-eosin and picro-Mallory staining, according to standard staining protocol.
