# Supplementary material for: A collateral circulation in ischemic stroke accelerates recanalization due to lower clot compaction
Source: PLoS One. 2024 Nov 19;19(11):e0314079. doi: 10.1371/journal.pone.0314079 (PMC11575800; doi:10.1371/journal.pone.0314079)
Supplement: S2 Note — (PDF) [file pone.0314079.s017.pdf]

S2 Note: Determination of clot length, relative clot reduction and clot degradation rate; Comparison of clot degradation rate *in vitro* with *in vivo* and patients

To compare clot degradation rate in *in vitro* model with *in vivo* and patients' data, clot length was determined at 30-minute intervals throughout the experiment course (time window 180 min) or until the release of the clot from the occluded *in vitro* vessel. Relative clot reduction was determined, expressing relative change in clot length just before release from the *in vitro* vessel; thus, without involvement of the information about recanalization time. To evaluate clot degradation in time-dependent manner, average clot reduction at time intervals was determined. In this case, clot length was counted as zero since the recanalization time, thus this expression interprets the combination of both parameters, thrombolysis and recanalization time.

In vivo and in patients, the change of clot area, instead of clot length, is usually determined due to the different diameters of occluded vessels between individual patients. Because the *in vitro* vessel has the same diameter in all models, the conversion factor (relative relation) between length and area is the same in all cases. Because of practical point of view, the method of determining the length was prioritized.
